# Non-psychotropic medication and risk of suicide or attempted suicide: a systematic review

**DOI:** 10.1136/bmjopen-2015-009074

**Published:** 2016-01-13

**Authors:** Hayley C Gorton, Roger T Webb, Navneet Kapur, Darren M Ashcroft

**Affiliations:** 1 Centre for Pharmacoepidemiology and Drug Safety, Manchester Pharmacy School, University of Manchester, Manchester, UK; 2 NIHR Greater Manchester Primary Care Patient Safety Translational Research Centre, University of Manchester, Manchester Academic Health Sciences Centre (MAHSC), Manchester, UK; 3 Centre for Suicide Prevention, Centre for Mental Health and Safety, Institute of Brain, Behaviour and Mental Health, University of Manchester, Manchester, UK

**Keywords:** EPIDEMIOLOGY, MENTAL HEALTH

## Abstract

**Objectives:**

To establish which non-psychotropic medications have been assessed in relation to risk of suicide or attempted suicide in observational studies, document reported associations and consider study strengths and limitations.

**Design:**

Systematic review.

**Methods:**

Four databases (Embase, Medline, PsycINFO and International Pharmaceutical Abstracts) were searched from 1990 to June 2014, and reference lists of included articles were hand-searched. Case–control, cohort and case only studies which reported suicide or attempted suicide in association with any non-psychotropic medication were included.

**Outcome measures:**

The outcomes eligible for inclusion were suicide and attempted suicide, as defined by the authors of the included study.

**Results:**

Of 11 792 retrieved articles, 19 were eligible for inclusion. Five studies considered cardiovascular medication and antiepileptics; two considered leukotriene receptor antagonists, isotretinoin and corticosteroids; one assessed antibiotics and another assessed varenicline. An additional study compared multiple medications prescribed to suicide cases versus controls. There was marked heterogeneity in study design, outcome and exposure classification, and control for confounding factors; particularly comorbid mental and physical illness. No increased risk was associated with cardiovascular medications, but associations with other medications remained inconclusive and meta-analysis was inappropriate due to study heterogeneity.

**Conclusions:**

Whether non-psychotropic medications are associated with increased risk of suicide or attempted suicide remains largely unknown. Robust identification of suicide outcomes and control of comorbidities could improve quantification of risk associated with non-psychotropic medication, beyond that conferred by underlying physical and mental illnesses.

**Strengths and limitations of this study**

- To our knowledge, this is the first systematic review to critically evaluate observational studies that have reported suicide and attempted suicide in relation to non-psychotropic medication.
- To reduce misclassification, which is problematic with other broader definitions of suicidality, only suicide and attempted suicide outcomes were considered.
- Study heterogeneity precluded statistical pooling of studies within each group of non-psychotropic medication via meta-analyses.

## Introduction

Worldwide, approximately 800 000 people die by suicide annually,^1^ therefore suicide prevention is an international priority.^2^ In addition to being the single strongest predictor of suicide,^1^ attempted suicide increases risk of all-cause mortality.^3^ A multitude of factors contribute to raised suicide risk,^4^ ^5^ in particular the presence of mental illness.^6^ ^7^ Additionally, the elevated risk of suicide associated with physical illnesses is becoming increasingly recognised,^7–9^ albeit to a lesser extent than the risk associated with mental illness.^7^

Although suicide risk differs between physical illnesses,^7^ individuals who have been hospitalised for any physical illnesses are at higher risk of suicide than those who have not.^8^ Various factors may contribute to increased suicide risk, including disease severity, comorbidities and impact on quality of life. Furthermore, it is largely unknown whether the non-psychotropic medications used to treat physical illnesses influence suicide risk beyond that attributed by the illness itself.

In 2009, the US Food and Drug Administration (FDA) required 125 medications, some non-psychotropic, to provide labelled warnings of suicidal ideation or behaviour, or both, in product information.^10^ Suicidality outcomes encompass a broad spectrum of suicidal intent, ranging from passive ideation without active planning to harm oneself, to self-harm without intent to die, to attempted suicide, to death by suicide.^11^ More commonly occurring suicidality outcomes are used as proxies for suicide and attempted suicide because randomised controlled trials (RCTs) are greatly underpowered to examine these rare outcomes Furthermore, participants of RCTs are closely monitored and study medication will be stopped if serious outcomes are observed. Therefore, assessment of suicide and attempted suicide in observational studies is essential to examine potential risks posed by non-psychotropic medication independent of the underlying physical illness.

Associations between selected non-psychotropic medications and suicidality have been considered in narrative reviews^12–14^ and one systematic review has focused on antiepileptic drugs (AEDs).^15^ However, to our knowledge, no systematic review which considers the extent of the associations between all non-psychotropic medications and suicide has been published. We therefore aimed to: (1) identify which non-psychotropic medications have been examined in relation to risk of suicide and attempted suicide in observational studies; (2) discern what associations have been reported; and (3) critically evaluate the strengths and limitations of these studies.

## Method

### Literature search

Four electronic databases, Embase, Medline, PsycINFO and International Pharmaceutical Abstracts*,* were independently searched. In all searches, there was a requirement for *suicide* or *suicidal* to be in the title or abstract. Terminology was selected to encompass any non-psychotropic medication. Psychotropic medications exert their main effect on mental symptoms^16^ therefore, non-psychotropic medication was accepted as medication not primarily prescribed to treat the mental illnesses described in *Diagnostic and Statistical Manual V*,^17^ and operationalised by exclusion of *British National Formulary* 68 categories 4.1–4.4, 4.10.1, 4.10.3 and 4.11.^18^ Medication search terms, medical subject headings and explode features were tailored for each database, and required presence in titles or abstracts. The following initial search strategy was used in Embase: suicide or suicidal (ti, ab) AND medicine (ti. ab.) OR medicat* (ti. ab.) OR drug therapy (exp., ti.ab.). Retrieved citations were limited to those published in English between 1990 and June 2014, to encompass any stimulated reporting following a case series of reports regarding suicidality published in 1990.^19^ For each medication group identified, additional searches were performed and reference lists of included studies were hand-searched. The full search strategy along with the study protocol is documented in the online supplementary material.

### Study inclusion

One author (HCG) screened studies against inclusion protocol and the other coauthors (DMA, RTW and NK) provided advice where a decision to include/exclude was unclear. Observational studies including cohort, case–control, case-crossover and self-controlled case series analyses, which pertained to any non-psychotropic medications, were eligible for inclusion. The outcomes of interest were suicide and attempted suicide presented separately or as a combined outcome. Other suicidality outcomes, including suicidal ideation, were excluded. Where authors indicated that the outcomes of interest were analysed separately, but outcomes were published only in combination with other suicidality outcomes, personal contact with these authors was made. Case reports, case series, cross-sectional studies, and RCTs were excluded. Any comparison treatment was permitted. Individuals with psychiatric illness were included providing the cohort was not defined by presence of this illness. This is because symptomatic improvement of the mental illness by medication used to treat the illness may preclude detection of any induction of suicidality and prevent equivalent comparison with non-psychotropic use. It was expected that AEDs would be a group of medicine retrieved by the literature search. This group does not feature as a class of clinical psychotropic medication per se, but some AEDs would also be classified as mood stabilisers, which are considered psychotropic.^16^ To avoid misrepresentation of the scope of non-psychotropic medication investigated in relation to suicide, we included AEDs in this systematic review. However, any study which focused on the use of AEDs exclusively as mood stabilisers was excluded.

### Study analysis

Study characteristics, key findings (eg, odds ratios, relative risks) and a critical appraisal, including an assessment of bias, are reported for each study in accordance with the *Preferred Reporting Items for Systematic Reviews and Meta-analyses* (PRISMA) statement.^20^ Studies of all quality levels were included in the review and were critiqued by one author (HCG) and discussed at length with co-authors (DMA and RTW). Existing quality assessment tools do not specifically relate to pharmacoepidemiological studies therefore, the criteria outlined in Neyarapally *et al*'s^21^ quality assessment framework was used to guide the critical evaluation.

## Results

**Figure 1:**
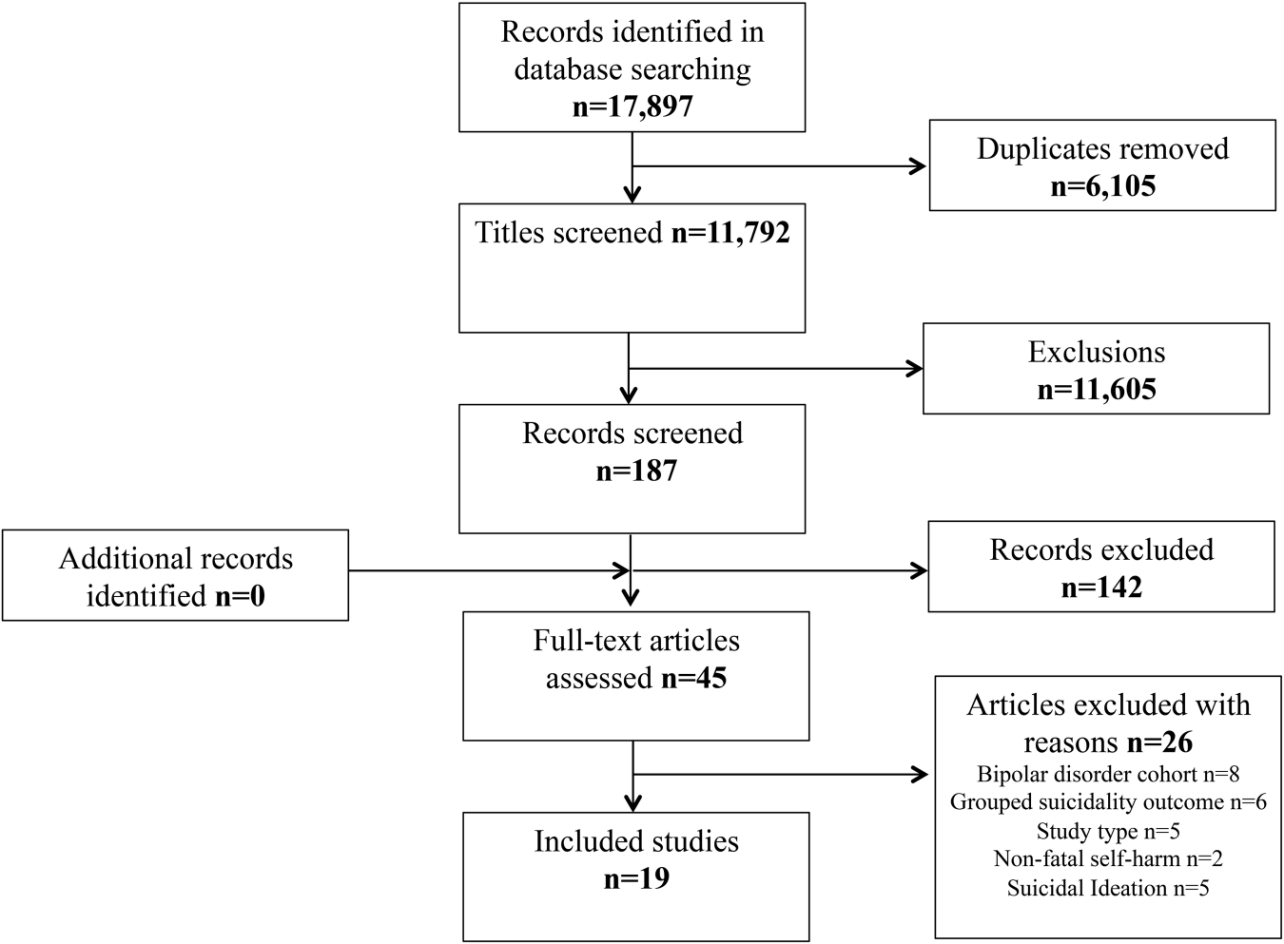
Flow diagram of included studies.

From 11 792 retrieved articles, 19 empirical studies (figure 1) satisfied our inclusion criteria*.* A primary focus on psychotropic medication, non-relevant outcomes or alternative study design, accounted for the majority of exclusions. Of the included studies (table 1) five studies each explored AEDs^22–26^ and cardiovascular medications^27–31^ two studies each considered leukotriene receptor antagonists (LTRAs),^32^ ^33^ isotretinoin,^34^ ^35^ and corticosteroids;^36^ ^37^ and one each assessed antibiotics^38^ and varenicline.^39^ One additional study compared various medications used by individuals who died by suicide, to those used by age and sex-matched controls.^40^

**Table 1:** Characteristics and critique of included studies

			Outcome definition					
Study ID and design	Participants	Exposure	Suicide	Attempted suicide	Combined suicide and attempted suicide	Outcome measures (95% CI)	Factors adjusted for in statistical analysis	Critique of study
Antiepileptic drugs								
Arana *et al*;^22^ cohort and case–control	THIN, UK, 1/7/1988–31/03/2008; cohort n=5 130 795; first suicide event n=8212 (completed n=464, attempted n=7748); case–control: any suicide event cases n=10 164, controls n=51 005	AED (carbamazepine/gabapentin/lamotrigine/levetiracetam/oxcarbazepine/pregabalin/tiagabine/topiramate/valproate/zonisamide)	–	–	Read codes (unspecified) for suicide, attempted suicide and intentional self-inflicted injuries plus suicide. Suicide death determined if code for death in month following suicidality code, and final database activity within 6 months	Cohort study: described incidences Case–control study: adjusted OR vs no epilepsy/bipolar/depressive disorder and no AED No epilepsy/bipolar/depressive disorder with AED: 2.57 (1.78 to 3.71) Epilepsy with AED: 2.31 (1.77 to 3.02); Epilepsy and no AED: 3.34 (2.34 to 4.78) OR AED use in epilepsy vs non-use: 0.59 (0.35 to 0.98)	Age, disease duration, history: AED/antidepressant/lithium/antipsychotic/mental illness/alcohol abuse; chronic disease score Excluded: personal or family history of suicide attempt	Grouped outcomes: AEDs grouped and proportions of individual AEDs not presented
Gibbons *et al*;^23^ cohort	PharMetrics Patient Centric Database, USA, 2000–2006. Cohort n=131 178; suicide attempt before initiation n=456, suicide attempt after initiation n=453	Gabapentin	–	ICD-9 codes E950–959	–	Adjusted event rate ratio after gabapentin initiation vs before initiation: epilepsy: 0.83 (0.34 to 2.04); pain disorder: 0.99 (0.78 to 1.26) Gabapentin monotherapy: 0.53 (0.16 to 1.73)	Adjustments: age, sex, concomitant diagnosis/treatment. Stratified by conditions.	Few outcomes in epilepsy and gabapentin monotherapy groups
Nilsson *et al*;^24^ case–control	Stockholm County Council In-Patient Care Register, age>15; epilepsy diagnosis and inpatient 1980–1989. Cases: death before 31/12/1997; age <78; controls: alive on 31/12/1992 n=171 suicide and undetermined suicide n=49 (n=26 in analyses)	Controls: phenytoin/carbamazepine/valproate Cases: any AED	ICD-9 E950–959; ICD-10 X60-X84, Undetermined intent: 980–989; Y10–34	–	–	Adjusted relative risk vs 1 AED:2 AED: 2.0 (0.8 to 5.2); 3 AED: 3.1 (0.6 to 17.5) Relative risk: number of dose changes vs 0 dose changes: 1–5 changes: 1.2 (0.4 to 3.4); unknown number of dose changes: 13.6 (3.8 to 49.2)	Age, sex	No adjustment for psychiatric comorbidities. Cases and controls unmatched but similar distributions seen. Controls subject to immortal time bias because^61^ AED use required for ≥1 year. Few cases may affect statistical power
Olesen *et al*;^25^ case-crossover analyses and cohort	Danish databases: National Prescription Register, Danish National Patient Register and National Causes of Death Registry 1/1/1997–31/12/2006, age ≥10. Case-crossover: suicide total n=6780, in study period n=898. Cohort: newly prescribed AED n=169 725, Suicide n=670 during treatment n=268	AED (carbamazepine/clobazam/clonazepam/gabapentin/lamotrigine/levetiracetam/ oxcarbazepine/phenobarbital/ phenytoin/pregabalin/ primidone/ tiagabine/topiramate/valproate/zonisamide)	National Cause of Death Register: ICD-10 X60-X84	–	–	Case-crossover analyses: OR AED exposure in case period vs control period: Overall AED: 1.84 (1.36 to 2.49); carbamazepine: 0.48 (0.21 to 1.12); gabapentin: 2.20 (0.83 to 5.83); lamotrigine: 3.15 (1.35 to 7.34); oxcarbazepine: 0.84 (0.30 to 2.32); phenobarbital 1.96 (1.02 to 3.75); valproate: 2.08 (1.04 to 4.16); topiramate: 2.72 (0.23 to 32.78) Cohort Study: adjusted HR AED initiation vs carbamazepine: gabapentin:1.27 (0.66 to 2.44); lamotrigine: 2.09 (1.25 to 3.50); oxcarbazepine 1.69 (0.81 to 3.56); valproate: 2.40 (1.42 to 4.05)	Cohort analyses: age, sex, socioeconomic status, Charlsons’ score, civil status, epilepsy/psychiatric disorder, opiate <180 days prior to index date; concomitant antidepressant/antipsychotic/anxiolytic	Case-crossover design suitability: Exposure may be influenced by indication which may independently increase risk of suicide when exposed to treatment
Patorno *et al*;^26^ cohort	HealthCore Integrated Research Database, USA; 07/2001–12/2006, age ≥15; new AED Cohort n=297 620 new treatment episodes, Suicide n=26, Suicide Attempt n=801	New exposure to AED (carbamazepine/ethosuxamide/felbamate/gabapentin/lamotrigine/levetiracetam/oxcarbazepine/phenobarbital phenytoin/pregabalin/primidone/tiagabine/topiramate/valproate/zonisamide)	–	–	Suicide attempt: emergency department/hospitalisation ICD-9 E950-E958 Suicide: ICD-10 X60–84	Adjusted HR for suicide and suicide attempt within 180 days of exposure vs topiramate: carbamazepine: 1.24 (0.77 to 1.99); gabapentin: 1.42 (1.11 to 1.80); lamotrigine: 1.84 (1.43 to 2.37); levetricetam: 1.63 (0.84 to 3.16); oxcarbazepine: 2.07 (1.52 to 2.80); valproate: 1.65 (1.25 to 2.19) Propensity score matched HR for suicide or suicide attempt in epilepsy/seizure disorder stratum vs carbamazepine: gabapentin: 13.92 (1.82 to 106.38); oxcarbazepine: 0.73 (0.16 to 3.28); phenytoin: 3.48 (0.97 to 12.47); topiramate: 0.67 (0.37 to 1.19); valproate: 0.49 (0.09 to 2.70)	49 covariates including diagnosis of or medication for: depression, mania, psychosis, anxiety, substance/alcohol abuse, personality disorder, other psychiatric disorders, physical disorders Propensity score matched analysis: in sensitivity analysis	Comparator suitability: topiramate selected but low frequency of use in epilepsy, although comparison with carbamazepine was repeated for this stratum. Imprecise estimate for gabapentin when restricted to people with epilepsy/seizure disorder: requires cautious interpretation
Cardiovascular drugs								
Callréus *et al*;^27^ Nested case–ontrol	Danish Registry of Cause of Death and The Odense University Pharmacoepidemiological Database, 1991–1998. Suicide Cases n=743, controls n=14 860	Medications used for lipid lowering, CCB, β-blockers, ACE-I and ARIIB	ICD-8 E950–959, ICD-10 X60-X84, Y87	–	–	OR for current use vs no use: statin: 1.25 (0.42 to 3.76); any lipid lowering drug: 1.21 (0.45 to 3.28); CCB: 0.96 (0.63 to 1.48); β-blocker: 0.76 (0.47 to 1.25); ACE-I: 1.11 (0.68 to 1.83); ARIIB: 3.52 (1.33 to 9.30)	Adjusted for: ever use of antipsychotics, lithium, antidepressants and drugs for alcohol dependence; use of antidiabetics or AED in past year. Sensitivity analysis: excluded if history of psychotropic drug use	Limited statistical power for some strata
Gasse *et al*;^28^ Nested case–control	GPRD, UK, 01/1991–08/1998, cases n=38 controls n=140 nested in cohort of individuals with hypertension diagnosis and prescription for antihypertensive medication	Antihypertensive (including CCB, β-blocker, ACE-I, diuretic)	OXMIS 3009D	–	–	Adjusted relative risk CCB vs other antihypertensive: 0.98 (0.30 to 3.18)	Body mass index, smoking and history of mental health illness	Potential under ascertainment of suicide outcomes by use of OXMIS codes but this is likely to be non-differential (N.B. at time of publication outcome definition suitable)
Haukka *et al*;^29^ cohort	Finish databases: Social Insurance Institution, National Hospital Discharge Register, Causes of Death Register; 01/01/1997–31/12/2005. Exposed n=336 618; Unexposed n=336 618; Suicide n=350	Statin	Cause of Death Register: ICD-10 X60-X84	–	–	Adjusted HR statin exposed vs unexposed: 0.53 (0.43 to 0.65) Poisson regression analysis of suicide per years of follow-up in statin use vs non-use: 0.5 ≤1 year: 0.49 (0.20 to 1.16);1 ≤2 years: 0.59 (0.28 to 1.25); 2 ≤3 years: 0.26 (0.11 to 0.60); 3 ≤4 years: 0.50 (0.21 to 1.19)	Adjusted: Sex, age, baseline diseases Additional adjustments in Poisson model: follow-up time, statin group Excluded: history of antidepressant medication	Limited adjustment for confounding factors: particularly psychiatric comorbidity (although individuals with depression excluded). Not generalisable to individuals with depression
Lindberg *et al*;^30^ cohort	Swedish pharmacy data,1988–1989, cohort n=3397; CCB n=617, other cardiovascular medication n=2780	Cardiovascular medication including CCB, β-blocker, ACE I, diuretic	Swedish Mortality Register: ICD-9 E950–959, E980–989	–	–	7-year suicide risk difference for CCB use vs non-use: 7.5/1000 person-years (p=0.002) Adjusted relative risk: 5.4 (1.4 to 20.5)	RR adjusted for age and sex.	Limited statistical power: 9 suicides in total. No adjustment for many confounders: including history of mental illness. Indication unknown
Sørensen *et al*; 31 cohort	Pharmacoepidemiological Prescription database of North Jutland County, Denmark, 01/01/1989–31/12/1995; cohort n=58 529	CCB, β-blocker or ACE inhibitor	National Death Certificate Files: ICD 8/10 codes	–	–	SMR ever-use: β-blocker: 1.6 (1.2 to 2.1); CCB: 1.2 (0.8 to 1.7); ACE-I 1.2 (0.7 to 1.8). SMR present use of single study drug: β-blocker: 1.4 (0.9 to 2.1); CCB: 1.2 (0.7 to 1.9); 1.2 (0.5 to 2.4). SMR for β-blocker use only (present and former): low lipid solubility: 0.9 (0.4 to 1.9); high lipid solubility: 2.7 (1.7 to 4.1)	–	Comparison to general population: differences in baseline characteristics and adjustment for confounders not possible (although some potential confounding factors compared between treatment groups). Potential overestimation of exposure: present use considered for 180 days after prescription received
Leukotriene receptor antagonists								
Jick *et al*;^32^ cohort	GPRD, UK, 02/1998–03/2007; cohort n=23 500	Montelukast	Computer recorded diagnosis	–	–	Suicides in 21 050 person years n=0		No comparison group-would be necessary to compare incidence if cases were identified
Schumock *et al*;^33^ Nested case–control	LifeLink Health Plan Claims Database, USA, 1/1/1997–31/12/2006, asthma and new use of an asthma treatment; age 5–24. Cases n=344 Controls n=3438	Montelukast, zafirlukast, zileuton	–	ICD-9 code E950-E959	–	Adjusted OR vs never use: current use: 0.70 (0.36 to 1.39), immediate past use: 0.95 (0.36 to 2.50), past use: 0.69 (0.32 to 1.50). Ever use: 0.74 (0.46 to 1.20)	History: bipolar disorder, depression, other mental disorder, substance abuse, suicide attempt; psychological counselling; asthma severity (by proxy)	All cases were exposed to montelukast rather than other drugs in this class (reflects prescribing trends), but results attributed to entire class
Isotretinoin								
Jick *et al*;^34^ cohort	Canadian Saskatchewan Health Database,1983–1997, acne diagnosis and drug exposure; isotretinoin n=7195, antibiotic n=13 700; suicide or SA n=38 GPRD: data not analysed	Isotretinoin or antibiotic (erythromycin/ doxycycline/ minocycline/tetracycline)	–	–	ICD-9 E codes	Adjusted relative risk vs non-exposure: isotretinoin: current use: 0.9 (0.3 to 2.4); recent use: 1.1 (0.2 to 3.7) Antibiotic: current use: 0.8 (0.4 to 1.7); recent use: 0.5 (0.1 to 1.4) Adjusted relative risk vs non-exposure in individuals with no psychiatric history: Isotretinoin: current use: 1.3 (0.3 to 4.6); recent use: 1.0 (0.1 to 5.7) Antibiotic: current use: 0.5 (0.1 to 1.6); recent: 0.7 (0.1 to 2.7)	Adjusted for sex, history of psychiatric disorder (depression, psychosis, attempted suicide) Stratified: by history of psychiatric disorder	Stratified analysis of individuals with no psychiatric history: small number may limit power.
Sundström *et al*;^35^ cohort and crossover analysis	Patient Register (mandatory), Sweden; 1980–1989 (outcomes identified until 2001), aged 15–49, cohort: n=5756; suicide: n=24; suicide attempt: n=128	Isotretinoin	National cause of death registry (including unclear intent), ICD codes	Suicide attempt hospitalisation ICD-8 and 9 E950-E958, E980-E988: ICD-10 X60–64; Y10-Y34	–	Cohort: standardised incidence ratio for all suicide attempts: isotretinoin users vs general population: 3 years pre-treatment: 0.99 (0.65 to 1.44); 1 year pretreatment: 1.57 (0.86 to 2.63); <6 months post-treatment: 1.78 (1.04 to 2.85); 3 years post-treatment: 1.04 (0.74 to 1.43) Case-crossover: rate difference 1 year pretreatment vs 6 months post-treatment: 1st attempts: 0.86 (−0.78 to 2.50) cases/1000 person-years; all attempts: 0.40 (−1.46 to 2.26) cases/1000 person-years SMR for suicide: incompletely recorded	–	Outcome misclassification possible: attribution of suicide attempt to exposure for up to15 years following exposure, may overestimate attempts. Comparison with general population: no control for confounding factors, including confounding by indication. This was explored in the case-crossover design
Corticosteroids								
Fardet *et al*;^36^ cohort	THIN, UK, 01/01/1990–31/12/2008; age ≥18; new glucocorticoid exposure n=372 696; exposed with indication n=261 272; unexposed n=1 224 984; unexposed matched by indication n=660 776; exposed groups suicide n=19; suicide attempt n=90	Oral glucocorticoid (betamethasone/deflazacort/dexamethasone/hydrocortisone/methylprednisolone/prednisone/prednisolone/triamcinolone/)	–	–	Read codes and cross-searched death certificates	Adjusted HR: exposed vs unexposed: 5.27 (3.82 to 7.29) Exposed vs unexposed, matched by indication: 6.89 (4.52 to 10.50)	Adjusted: age, sex, history neuropsychiatric disorder Separate analysis for cohorts matched by indication	Handling of repeated courses: random selection of course could alter baseline risk dependent on course selected. If later courses chosen, individual subject to immortal time^61^ bias until this time
Fardet *et al*;^37^ cohort and self-controlled case series	THIN, UK 01/01/1990–31/12/2008; age≥18; glucocorticoid use for 1–3 years. Cohort: n=21 995. Eligible for self-controlled case series analysis: n=991; suicide or suicide attempt n=6	Oral glucocorticoid (betamethasone/deflazacort/dexamethasone/hydrocortisone/methylprednisolone/prednisone/prednisolone/triamcinolone/)	–	–	Read codes	Cohort: incidence rate for suicide or attempted suicide during withdrawal period: 0.03 (0.01 to 0.2) Self-controlled case series: incident rate ratio in withdrawal period vs ref. period: 0.62 (0.06 to 6.92) (ref. period:5–3 months prior to discontinuation)		Inadequate statistical power: only 6 cases of suicide or attempted suicide. Potential immortal time bias:^61^ for entry into the cohort, must not have died in first year of glucocorticoid use. To be eligible for the self-controlled case series analysis, any suicides must occur in the withdrawal period
Antibiotics (quinolones)								
Jick *et al*;^38^ Nested ase Control	GPRD, UK, ever exposed to quinolone, 01/01/1991–30/4/1995, age 15–84. Cases n=348 (suicide n=13, suicide attempt n=206 suicidal ideation n=129) Control n=808 (NB. Outcomes analysed separately)	Quinolone or other antibiotic in 1–30 or 31–180 days prior to index date	OXMIS code 3009D	OXMIS code L3009P, 9779 L, 3009C	–	Adjusted relative risk estimate for suicide attempt vs non-exposure: quinolone 1–30 days: N/A; quinolone 31–180 days: 0.6 (0.2 to 1.5); other antibiotic 1–30 days: 1.2 (0.5 to 2.6); other antibiotic 31–180 days: 0.9 (0.5 to 1.5)	Age, sex, history of: depression, suicidal behaviour, insomnia, psychosis, anxiety, alcoholism and epilepsy	Inadequate statistical power: precluded calculation of risk within first month of quinolone exposure or risk of suicide death. Possible underestimation of suicide and attempted suicide: because 1st recorded event used in multiple outcomes. However, all suicidal ideation comparisons were non-significant
Varenicline								
Gibbons and Mann;^39^ cohort	Military Healthcare System, USA, 01/08/2006–31/08/2007. Cohort treated with varenicline (n=19 933); NRT patches (n=15 867). After matching by propensity score: patients n=26 430; included suicide attempts n=5	Varenicline or NRT patches	–	ICD-9 E950–959	–	OR varenicline vs NRT in patients for whom propensity score matching was possible: 0.67 (0.11 to 3.99)	Propensity score matching: age, marital status, race, sex, Charlsons’ score, inpatient admissions, psychiatric comorbidity, psychotropic medication	Limited power: few suicide attempt events (n=5)
Other medication								
Voaklander *et al*;^40^ case–control	British Columbia Vital Statistics, Health Insurance Registration File, Pharmacare and Physician Claim File, Canada, 1993–2002, age ≥66, suicide cases n=602 controls n=2999	anti-diabetic agents, anticoagulants, cardiovascular drugs, NSAID, ulcer medication, steroids	ICD-9 E950-E959; ICD-10: X60—X84	–	–	Unadjusted ORs vs non-use: antihypertensive medication: 0.94 (0.67 to 1.31); lipid lowering medication: 0.60 (0.28 to 1.26); anticoagulants: 1.07 (0.52 to 2.22); diuretics: 0.94 (0.66 to 1.36); ulcer medication: 1.88 (1.35 to 2.62); steroids: 1.33 (0.88 to 2.00). Fully adjusted OR: diuretics: 0.49 (0.31 to 0.76)	Fully adjusted analyses: demographics, co-morbidity, medication use	Fully adjusted analysis not done for all medications which suggested significance at the unadjusted level (eg, ulcer medications)

AED, antiepileptic drugs; ARIIB, angiotensin receptor II blocker; CCB, calcium blocker; CNS, central nervous system; GPRD, general practice research database; ICD, International Classification of Diseases; NRT, nicotine replacement therapy; NSAID, non-steroidal anti-inflammatory drug; OXMIS, Oxford Medical Information System; SMR, standardised mortality ratio; THIN, The Health Improvement Network.

Nine studies reported suicide,^24^ ^25^ ^27–32^ ^40^ three reported attempted suicide,^23^ ^33^ ^39^ two studies presented outcomes separately^35^ ^38^ and five of them combined these outcomes.^22^ ^26^ ^34^ ^36^ ^37^ Some studies linked suicide cases to national^24–27^ ^29–31^ or local^40^ mortality data, and others relied on database coding of suicide and attempted suicide.^22^ ^23^ ^28^ ^32–34^ ^36–39^ Studies were conducted in the UK,^22^ ^28^ ^32^ ^36–38^ USA,^23^ ^26^ ^33^ ^39^ Canada^34^ ^40^ and Scandinavia,^24^ ^25^ ^27^ ^29–31^ ^35^ and therefore were subject to suicide recording conventions adopted by each country. Population sources included healthcare databases which recorded drugs prescribed,^22^ ^28^ ^32^ ^36–38^ dispensed,^27^ ^30^ ^31^ or both;^23^ ^25^ ^26^ ^29^ ^33^ ^34^ ^39^ ^40^ and hospital inpatient^24^ or specialist registries.^35^

Thirteen studies accounted for psychiatric comorbidities to various extents.^22^ ^23^ ^25–29^ ^33^ ^34^ ^36^ ^38^ ^39^ ^40^ Statistical adjustment was the most commonly used method.^23^ ^25^ ^26–28^ ^33^ ^36^ ^38^ ^40^ Exclusion of patients with history of depression^29^ or suicide attempt,^22^ ^26^ stratification by psychiatric history^34^ and propensity score matching^26^ ^39^ were also used. In the studies which attempted to mitigate confounding by indication, medication use was restricted to particular conditions,^24^ ^28^ ^32–35^ stratified by condition^22^ ^23^ ^36^ or adjustment for physical illness was performed.^22^ ^25^ ^26^ ^40^ Some studies quantified suicide risk by comparison of a treated group with an untreated group,^22^ ^27^ ^29^ ^36^ the general population^31^ ^35^ or a group using other medications relevant to that condition.^26^ ^34^ ^39^ Use of an individual as their own control in case-only designs^25^ ^35^ ^37^ relinquished the need for a separate comparator group.

### Antiepileptic drugs

Of the five studies which investigated AEDs, two reported suicide,^24^ ^25^ one estimated risk of attempted suicide^23^ and two combined both outcomes.^22^ ^26^ Four studies utilised a cohort design,^22^ ^23^ ^25^ ^26^ one of which also performed a case-crossover analysis.^25^ Additionally, Arana *et al*^22^ utilised a case–control study, the only design used by Nilsson *et al*.^24^ Some studies considered individual AEDs,^23^ ^25^ ^26^ whereas others assessed all AEDs combined.^22^ ^24^ Comparisons were made with non-exposure,^22^ particular AEDs^25^ ^26^ or multiple AEDs compared to monotherapy in individuals diagnosed with epilepsy.^24^

The association between AEDs and suicide remains undetermined and varies between individual AEDs. Arana *et al* reported an increased risk of suicide and attempted suicide when AEDs were used for conditions other than epilepsy, bipolar disorder or depression; compared to controls who did not receive AEDs nor had these diagnoses (OR 2.57 (95% CI 1.78 to 3.71)). Conversely, within the epilepsy strata, a reduced risk was identified in the treated group compared to the untreated group (OR 0.59 (95% CI 0.35 to 0.98)).^22^

Three studies considered risk attributed to individual AEDs. Patorno *et al*^26^ suggested an increased risk of suicide and attempted suicide associated with gabapentin compared to topiramate (HR 1.42 (95% CI 1.11 to 1.80)).^26^ Conversely, both Gibbons *et al* (2010) 23 and Olesen *et al*^25^ reported no statistically significant difference in suicide attempt rate before or after gabapentin initiation. Patorno *et al*^26^ reported an increased risk of suicide and attempted suicide with valproate and lamotrigine compared to topiramate. However, when compared to carbamazepine in a cohort of people with epilepsy, no elevation in risk was identified whereas Olesen *et al*^25^ suggested an increased risk of suicide for the same AEDs, when indication was not restricted.

### Cardiovascular medications

Two nested case–control studies^27^ ^28^ and three cohort studies^29–31^ assessed risk of suicide associated with various cardiovascular medications. In all but one, there was no evidence of association with increased or decreased suicide risk. An initial suggestion of increased risk of suicide with calcium channel blockers (CCB) was made by Lindberg *et al* but the reported association was questioned due to small sample size and lack of control for confounding factors.^41^ ^42^ Subsequent studies dismissed any association with CCB use and suicide.^27^ ^28^ ^31^ Similarly, there was no difference in risk for ACE inhibitor or β-blocker use compared to non-use^27^ during monotherapy versus the general population.^31^ An increased standardised mortality ratio was, however, suggested for highly lipid soluble β-blockers but attributed in part to use in migraine.^31^

An unexpected increased suicide risk with angiotensin II receptor antagonists was reported by Callréus *et al*^27^ (OR 3.52 (95% CI 1.33 to 9.30)), despite control of multiple potential confounding factors. However, few suicides were reported (current use n=5) and, when controlled for psychiatric history, this association became non-significant. Based on the same number of suicide cases, no association between statins and suicide was made. Corroboratively, Haukka *et al*^29^ suggested no increase in suicide risk in statin users versus non-users, in any follow-up time investigated. When cardiovascular medication use was compared to non-use by Voaklander *et al*^40^ only diuretics were suggested to significantly influence risk; a protective effect was suggested in the fully adjusted analysis (OR 0.49 (95% CI 0.31 to 0.76)).

### Leukotriene receptor antagonists

Two observational studies reported that no increased risk of suicide or attempted suicide was apparent when LTRAs, montelukast, zafirlukast and zileuton, were used for the treatment of asthma.^32^ ^33^ No suicides were reported in Jick *et al*'s^32^ cohort of individuals exposed to montelukast, although one case was retrospectively disqualified based on time lag between exposure and outcome. Similarly, Schumock *et al*^33^ did not detect any difference in suicide attempt during use of any LTRA compared to non-use in their nested case–control study of individuals diagnosed with asthma, aged 5–24 years.

### Isotretinoin

No difference in the combined risk of attempted and completed suicide was associated with isotretinoin or antibiotics, compared to non-exposure, in Jick *et al*'s^34^ cohort of individuals with acne, regardless of psychiatric history. Similarly, there was no significant difference in attempted suicide risk before treatment compared to 6 months after treatment, in Sundström *et al*'s^35^ crossover analysis. On the other hand, when compared to the general population, the highest elevated risk was observed in the first 6 months of treatment, but this risk was rising prior to medication initiation.

### Corticosteroids

No difference in suicide risk was associated with steroid use versus non-use by Voaklander *et al*.^40^ Conversely, in Fardet *et al*'s (2012) cohort study, a fivefold increased risk of suicide and attempted suicide was reported following glucocorticoid exposure, compared to non-exposure (HR 5.27 (95% CI 3.82 to 7.29)) but incidence was low.^36^ In a subsequent self-controlled case series, no difference was detected during withdrawal period compared to treatment periods,^37^ although this assertion was based on only six cases.

### Antibiotics

In the single nested case–control study which focused on quinolone antibiotics, no difference in risk of attempted suicide was detected following exposure to quinolones or other antibiotics, compared to non-exposure.^38^

### Varenicline

One cohort study reported suicide attempt, separately from other outcomes, in relation to varenicline use. There was no difference in risk during use of varenicline or nicotine replacement patches, when individuals were matched by propensity score.^39^

## Discussion

The primary aims of this systematic review were to establish which groups of non-psychotropic medications have been associated with suicide and attempted suicide in observational epidemiological studies; and to quantify the influence these medications have on this risk, beyond that conferred by underlying illness. Overall, the contribution of corticosteroids,^36^ ^40^ isotretinoin^34^ ^35^ and AEDs^22–26^ to risk of suicide and attempted suicide remains unresolved while there seems no increased risk associated with cardiovascular medications.^27–29^ ^31^ Neither the single studies which investigated quinolones^38^ or varenicline,^39^ nor the two which assessed LTRAs,^32^ ^33^ suggested an increased risk of suicidality.

With the exception of the cardiovascular medicines, all groups of medications identified in this review have been the subject of FDA or UK Medicines and Healthcare Products Regulatory Agency (MHRA) warnings. In 2008, the FDA warned of an increased risk of suicidal behaviour and ideation during AED use, following a meta-analysis of 199 placebo-controlled RCTs involving 43 892 patients. An overall OR of 1.80 (95% CI 1.24 to 2.66) was reported.^43^ This mainly reflected an increased risk of suicidal ideation and attempted suicide because only four suicides in total were reported across almost 200 trials. This emphasises the lack of statistical power of RCTs, and even meta-analysis of numerous trials, for examining an outcome as rare as death by suicide. Concerns were raised from medical and research communities regarding the reliability and impact of this warning. The risk was attributed as a class effect despite variation in risk associated with individual AEDs, there was potential for heterogeneity in outcome designation and there was an unexplained differential risk dependent on study location.^44^ ^45^ Our review of the observational work which followed is in corroboration with Ferrer *et al*'s^15^ review, which considered AEDs for any indication, that the association between dying by suicide and AEDs remains inconclusive.

Varenicline is associated with the highest level of warnings issued by the FDA. It may therefore be surprising that only one study which pertains to varenicline is included in this review. This is because no other observational studies considered only suicide or attempted suicide outcomes. Neither observational studies which considered all self-harm outcomes^46^ ^47^ nor a pooled analysis of RCTs which considered all suicidality outcomes suggested an increased risk associated with varenicline,^39^ compared to other smoking cessation treatments or placebo. The FDA black-box warning continues to be challenged by the manufacturers of varenicline, based on a meta-analysis of placebo-controlled RCTs.^48^ The FDA also warns of suicidal behaviour during use of isotretinoin and in 2014, an expert review in the UK could neither confirm nor discount an association of suicide with isotretinoin.^49^ Isotretinoin is one of three non-psychotropic medications on the list of the top 20 medicines most frequently associated with suicide in UK spontaneous reports.^50^ The other two medications, efavirenz and mefloquine,^50^ carry warnings for suicidal behaviour in their drug monographs.^18^ No observational studies reported suicide or attempted suicide outcomes for these medications. Warnings of psychiatric adverse events, including suicidal behaviour, exist for glucocorticoids in the UK, but this group does not feature in the list of FDA drugs linked with suicidal behaviour.^10^

This systematic review has found considerable heterogeneity among studies, which makes comparisons within and between medication groups difficult and quantitative meta-analysis inappropriate. All of the studies that considered cardiovascular medications reported suicide only. Conversely, there was variation in reported outcomes within classes of other medications. Some studies reported attempted suicide or combined suicide and attempted suicide outcomes, sometimes because suicide outcomes were too rare to enable detection of differences between groups. Furthermore, different comparison groups were chosen. Schumock *et al*^33^ controlled for confounding by indication and disease severity by restricting the comparator cohort to people with asthma who use controller medication. On the other hand, comparator group demographics could not be stipulated when standardised mortality rates were estimated.^31^

It is imperative that the underlying risk posed by physical illness as well as pre-existing mental illness or psychological distress is recognised when interpreting any elevation in risk associated with non-psychotropic medication. This is of particular importance for AEDs because epilepsy has been associated with a twofold increased risk of suicide compared to the general population.^51^ Furthermore, AEDs can be prescribed for a variety of physical and psychiatric conditions including bipolar disorder, which is associated with over a 17-fold increased risk of suicide.^7^ ^52^ In this review, articles were excluded if the study population was defined by presence of mental illness, to aid interpretation of possible associations with medication separately from to those conferred by the mental illness. We acknowledge that a limitation of this review is that some studies allowed AED use for any indication, which may have included mental illness. This was, however, accounted for by adjustment for or stratification by mental illness in those studies.

Additionally, medications may be used for alternative indications where first line treatment has failed. This could contribute to the increased suicide risk observed by Arana *et al*^22^ when AEDs were prescribed for conditions other than epilepsy, depression or bipolar disorder, much of which was suspected to be indicated for pain. Similarly, Sørensen *et al*^31^ attributed the increased risk associated with lipid soluble β-blockers in part to the higher prevalence of migraine in this group. Glucocorticoids are often introduced during disease relapse which could contribute to suicide risk, even when indication is controlled for.^36^ Likewise, the increased suicide risk identified prior to initiation of isotretinoin, could be a factor of acne severity.^35^

Observational studies are essentially useful for demonstrating associations rather than causation, although tentative inferences of causality can be put forward if there is robust evidence of concurrence with Bradford-Hill's criteria.^53^ One of his seven pillars of causality is the biological plausibility of the event, in this case a postulated adverse reaction. Adverse reactions to medication can be denoted as type A, an exaggerated effect of the pharmacology of the medication, or type B, usually an idiosyncratic event, often detected in postmarketing surveillance.^54^ Any observed elevation in risk of suicide could be a consequence of induced depression or occur independently. Potential pathways to suicidality have been suggested for some, but not all, medicines included in this review. Interference with γ-aminobutyric acid and glutamate may contribute to any observed link between AED usage and elevated suicide risk,^55^ but would differ between individual AEDs. Increased cortisol levels have also been linked to suicide.^56^ Therefore exogenously introduced glucocorticoids could confer similar effects. Reduced lipid levels have also been associated with increased suicide risk.^5^ Conversely, the included studies do not suggest increased risk with lipid-lowering medication use,^27^ ^29^ corroborative with earlier work by Yang *et al*.^57^

To our knowledge, this is the first systematic review to consider the impact of non-psychotropic medication use on risk of suicide and attempted suicide. Only suicide and attempted suicide outcomes were considered, to minimise outcome misclassification possible when other suicidality outcomes are used as proxies.^11^ ^13^ Determination of an individuals’ intent to die by suicide is challenging^58^ ^11^ therefore other terminologies may incorporate suicide attempt. For example, ‘non-fatal self-harm’ represents a continuum of suicidal and self-harm behaviours with varying motivations and intentions. To avoid overestimation of outcomes, studies were included only if authors explicitly used the label ‘suicide attempt’. This may have precluded inclusion of studies which reported attempted suicide as a composite outcome within another definition of suicidality, and is therefore a limitation of our review. We also acknowledge that screening of studies by a single author introduced the potential for selection bias. In an attempt to reduce this bias, other authors were consulted if selection was unclear and included records were hand-searched for suitable studies, which served as a cross-check.

Inclusion of studies from any country introduced further variation in suicide classification.^59^ In the UK, open verdicts are conventionally included in epidemiological suicide definitions, as most are deemed to be probable suicides that were not designated as such due to the high burden of proof required in coroners’ courts.^60^ In other countries, including the USA, open verdicts are generally not included in suicide case definitions. For example, Patorno *et al* separately reported violent deaths in their US population, up to 87% of which may be suicides. The trends identified were, however, similar to those for suicide and attempted suicide outcomes.^26^

Determining the cause of any observed increased risk, specifically as a result of mental or physical illness, the medication itself, or a combination of factors, represents a major challenge. Overall assessments are difficult to report due to variation between study outcomes, populations and control for psychiatric and physical comorbidities. Robust identification of suicidality outcomes and control of comorbidities is needed in future observational studies, particularly to investigate suicide risk in association with AEDs, isotretinoin and corticosteroids.
